# The effects of field strength on stimulated echo and motion-compensated spin-echo diffusion tensor cardiovascular magnetic resonance sequences

**DOI:** 10.1016/j.jocmr.2024.101052

**Published:** 2024-06-25

**Authors:** Andrew D. Scott, Ke Wen, Yaqing Luo, Jiahao Huang, Simon Gover, Rajkumar Soundarajan, Pedro F. Ferreira, Dudley J. Pennell, Sonia Nielles-Vallespin

**Affiliations:** a Cardiovascular Magnetic Resonance Unit, Royal Brompton Hospital, Guy’s and St Thomas’ NHS Foundation Trust, Sydney Street, London SW3 6NP, UK; b National Heart and Lung Institute, Imperial College, Dovehouse Street, London SW3 6LY, UK; c EPSRC Centre for Doctoral Training in Smart Medical Imaging, King’s College London and Imperial College London, 5th Floor Beckett House, 1 Lambeth Palace Road, London SE1 7EU, UK; d Department of Bioengineering, Imperial College London, Royal School of Mines, Exhibition Road, London SW7 2AZ, UK

**Keywords:** Diffusion tensor, Field strength, Cardiac microstructure, DTI, Healthy volunteers, Stimulated echo

## Abstract

**Background:**

In-vivo diffusion tensor cardiovascular magnetic resonance (DT-CMR) is an emerging technique for microstructural tissue characterization in the myocardium. Most studies are performed at 3T, where higher signal-to-noise ratio (SNR) should benefit this signal-starved method. However, a few studies have suggested that DT-CMR is possible at 1.5T, where echo planar imaging artifacts may be less severe and 1.5T hardware is more widely available.

**Methods:**

We recruited 20 healthy volunteers and performed mid-ventricular short-axis DT-CMR at 1.5T and 3T. Acquisitions were performed at peak systole and end-diastole using both stimulated echo acquisition mode (STEAM) and motion-compensated spin-echo (MCSE) sequences at matched spatial resolutions. DT-CMR parameters were averaged over the left ventricle and compared between 1.5T and 3T sequences using both datasets with and without the b_low_ reference data included.

**Results:**

Eleven (1.5T) and 12 (3T) diastolic MCSE acquisitions were rejected as the helix angle (HA) demonstrated <50% normal appearance circumferentially or the acquisition was abandoned due to poor image quality; a maximum of one acquisition was rejected for other datasets. Subjective HA map quality was significantly better at 3T than 1.5T for STEAM (p < 0.05), but not for MCSE and other DT-CMR quality measures were consistent with improvements in STEAM at 3T over 1.5T. When b_low_ data were excluded, no significant differences in mean diffusivity were observed between field strengths, but fractional anisotropy was significantly higher at 1.5T than 3T for STEAM systole (p < 0.05). Absolute second eigenvector orientation (E2A, sheetlet angle) was significantly higher at 1.5T than 3T for MCSE systole and STEAM diastole, but significantly lower for STEAM systole (all p < 0.05). Transmural HA distribution was less steep at 1.5T than 3T for STEAM diastole data (p < 0.05). SNR was higher at 3T than 1.5T for all acquisitions (p < 0.05).

**Conclusion:**

While 3T provides benefits in terms of SNR, both STEAM and MCSE can be performed at 1.5T. However, MCSE is unreliable in diastole at both field strengths and STEAM benefits from the improved SNR at 3T over 1.5T. Future clinical research studies may be able to leverage the wider availability of 1.5T CMR hardware where MCSE acquisitions are desirable.

## Graphical abstract

**Figure:**
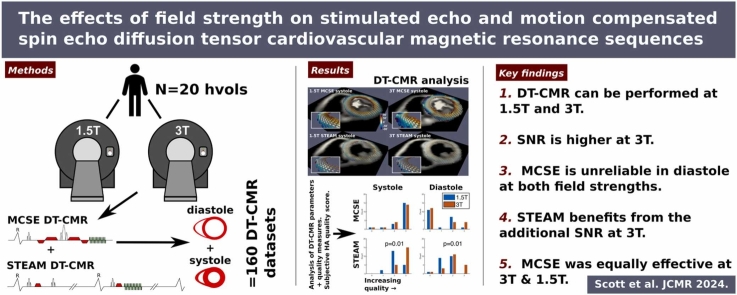

## 1 Background

In-vivo diffusion tensor cardiovascular magnetic resonance (DT-CMR) uniquely provides quantitative characterization of the myocardium based on the structure of the tissue at a microscopic level 1, 2, 3, 4, 5, 6, 7, 8. These sub-voxel insights are enabled by sensitizing the CMR signal to the distances diffused by the hydrogen nuclei, which are hindered and restricted by the membranes and other structures that they encounter on their random walk over the time they are observed. Typically, the three-dimensional distribution of diffusivity is modeled as a 3 × 3 tensor [9], where the primary eigenvector aligns with the mean intra-voxel cardiomyocyte orientation 3, 4, 10, 11. Obtaining the diffusion-weighted data to calculate the pixel-wise diffusion tensor in the presence of cardiac motion has been performed using either a stimulated echo acquisition mode (STEAM) sequence split over two cardiac cycles 2, 12, 13 or a second-order motion-compensated spin-echo (MCSE) sequence 5, 14. While both sequences are hindered by low signal-to-noise ratios (SNR), the STEAM sequence is inherently less SNR efficient than MCSE [15]. For either DT-CMR sequence, the images are most commonly acquired with a single-shot echo planar imaging (EPI) readout. While the MCSE sequence relies on high maximum gradient strengths to provide sufficient diffusion weighting during a reasonable echo time (TE), STEAM uses a pair of relatively small diffusion encoding gradients, placing them exactly one cardiac cycle apart, thereby allowing the water molecules ∼1 s to diffuse and generate a measurable level of diffusion weighting.

For both commonly used DT-CMR sequences, the vast majority of clinical and technical research has been performed at 3T 8, 16, 17, 18, where the greater difference in spin populations results in a theoretical increase in SNR at 3T over 1.5T by a factor of 2 (see Supplementary Material for a mathematical comparison). Furthermore, the T1 and T2 are shorter at 1.5T than 3T [19] and the TE may differ due to differences in hardware performance. While the increased T1 at 3T results in a reduction of the magnetization available for imaging due to incomplete recovery between sequence repeats for MCSE, for the STEAM sequence, the signal loss due to incomplete longitudinal recovery between sequence repetitions at 3T is balanced by the reduction in signal loss during the mixing time. In addition to SNR considerations, artifacts may be less severe at lower field strengths, the capital outlay of a 1.5T scanner is less and there are more of these systems available [20].

Here, we aim to compare the quality and identify any systematic differences in the microstructural parameters obtained from in-vivo DT-CMR between 1.5T and 3T for both STEAM and MCSE in multiple cardiac phases.

## 2 Methods

### 2.1 Cohort

We recruited 20 healthy subjects without a history of heart disease and without contraindications for DT-CMR (e.g. arrhythmia, implanted devices, cardiothoracic surgical clips) with written informed consent according to local ethical approval (National Research Ethics Service 10/H0701/112, approved October 2018).

### 2.2 Imaging protocol

All subjects were imaged at 1.5T (Magnetom Sola, Siemens, Erlangen, Germany, maximum gradient strength = 45 mT/m, maximum slew rate = 200 T/m/s) and 3T (MAGNETOM Vida, Siemens, Erlangen, Germany, maximum gradient strength = 60 mT/m, maximum slew rate = 200 T/m/s) using similar flexible anterior and spine array coils and identical software baselines.

Single mid-ventricular DT-CMR was planned based on multiple long-axis–balanced steady-state free precession cine data and a stack of short-axis slices. Peak systole and diastasis were identified from cine data in the selected short-axis plane and the imaging plane was manually tracked between the two cardiac phases based on vertical and horizontal long-axis cines with linear tagging applied parallel to the mitral valve [21]. DT-CMR was then acquired in this plane using STEAM and MCSE with the phase encode direction optimized on a subject/field strength/sequence/cardiac phase-wise basis by trial and error. Trigger delays were set separately for MCSE and STEAM in each scan for each subject to acquire the central k-space data at peak systole or to place the diffusion encoding and data acquisition during the most stationary period of diastasis. The slice position was matched between the acquisitions on the two scanners by measuring the distance from the mitral valve to the imaging plane and comparing long and short-axis images from the first acquisition.

### 2.3 DT-CMR sequences

DT-CMR data acquisition source code was identical between scanners. STEAM and MCSE EPI data were acquired during breath holding. All DT-CMR data were acquired with repetition time (TR) = 2RR intervals, six encoding directions + b_low_ reference data per breath hold (with an additional four RR-intervals used to acquire EPI phase correction and parallel imaging reference data), resulting in a breath hold duration of 18 cardiac cycles. The readout field of view was 360 × 135 mm^2^, 8 mm slice thickness, no partial Fourier, sensitivity encoding factor 2, acquired resolution 2.8 × 2.8 mm^2^ reconstructed using the manufacturer supplied reconstruction code at 1.4 × 1.4 mm^2^, fat saturation, EPI echo spacing 0.51 ms, and echo train length 23 lines. The echo-generating field of view was reduced in the phase encode direction by making the 90° pulse (MCSE) or the first two 90° pulses (STEAM) slice selective in the phase encode direction. TE is highly dependent on maximum gradient strength for MCSE and TE = 62 ms at 3T and TE = 74 ms at 1.5T. For STEAM TE = 25 ms at 3T and TE = 26 ms at 1.5T.

For all acquisitions, a reference b-value of 150 smm^−2^ was used and for all MCSE sequences a main b-value of 450 smm^−2^. For STEAM sequences, the main b-value was 600 smm^−2^ at 3T but was reduced to 450 smm^−2^ at 1.5T due to initial tests showing visually reduced SNR at the lower field strength. Due to the presence of spoiler gradients, the b_low_ reference data had an effective b = 34 smm^−2^ for STEAM sequences at both field strengths (hence we avoid using the term “b0”) and for MCSE sequences b = 0.13 smm^−2^ at 1.5T and b = 0.15 smm^−2^ at 3T. For both sequences, spoiler gradients are applied along all image encoding directions. The contribution of non-diffusion encoding gradients was confirmed to result in an error in the b-value of <1 smm^−2^. For each sequence and cardiac phase, eight repetitions (breath holds) were acquired at the main b-value and two repetitions (breath holds) were acquired at b = 150 smm^−2^, meaning that the number of breath holds and breath-hold duration was matched between all combinations of sequence, cardiac phase and field strength.

### 2.4 Analysis

All DT-CMR data were processed using an in-house MATLAB (The MathWorks, Natick, Massachussets) software tool [21]. Motion-corrupted frames were rejected manually, and diffusion-weighted images were registered for each sequence and cardiac phase using a non-rigid b-spline algorithm (Elastix 22, 23), before segmenting the epi- and endocardium of the left ventricle (LV) and calculating the diffusion tensor at every pixel from the magnitude images using an iterative non-linear least squares method. All images were included in the tensor calculation without averaging. For STEAM data, the b-value used in tensor calculation was corrected for the effect of the RR interval on diffusion weighting [24]. The helix angle (HA), absolute angle of the second eigenvector (E2A, see [21]), transverse angle (TA), mean diffusivity (MD), and fractional anisotropy (FA) were calculated from the tensor. Using a linear fit to profiles of the HA from epicardium to endocardium, the HA gradient (HAG) in degrees per percent wall thickness was calculated.

Datasets were each processed twice, once including all b-values and then with the b_low_ reference images removed using the same registration and regions of interest in both cases. While our previous work has suggested that using a reference b ≈ 150 smm^−2^ rather than the b_low_ reference data can reduce the effects of perfusion on the calculated tensor [24], this does reduce the amount of data used in the tensor calculation and previous studies have used both approaches. For completeness, we include the results of both analyses here, providing reference values for use by the DT-CMR community.

To assess the quality of the DT-CMR data acquired at 1.5T and 3T, HA maps were qualitatively scored [25] based on the visual proportion of the myocardium demonstrating the expected change of HA from epi to endocardium. A score of 0 for <50%, 1 for 50%–75%, 2 for 75%–95% and 3 for >95%. Data scoring 0 were excluded from further analysis.

SNR maps were calculated using the multiple repetitions method [26] for comparison between field strengths. For the STEAM data, SNR was calculated for the b_low_ data, while for the MCSE data, SNR was calculated for the b = 450 smm^−2^ data (then averaged over the six encoding directions) due to unsuppressed blood signal in the b_low_ data for MCSE. Other quality metrics used were the proportion of LV pixels with one or more negative eigenvalues, the standard deviation of the TA, and the Pearson R^2^ of the linear fit to the transmural HA data (where we exclude HA profiles with a positive slope and an R^2^ < 0.3).

Parameters were averaged over the LV (mean or median for E2A). Statistical analysis was performed in MATLAB. Parameters were tested for normality over the subject cohort using a Shapiro-Wilks test. Parameters were compared between field strengths for each cardiac phase and sequence type using a t-test (for normally distributed data) and a Wilcoxon-sign rank test otherwise or for ordinal, non-continuous data. p < 0.05 was considered significant.

## 3 Results

**Fig. 1:**
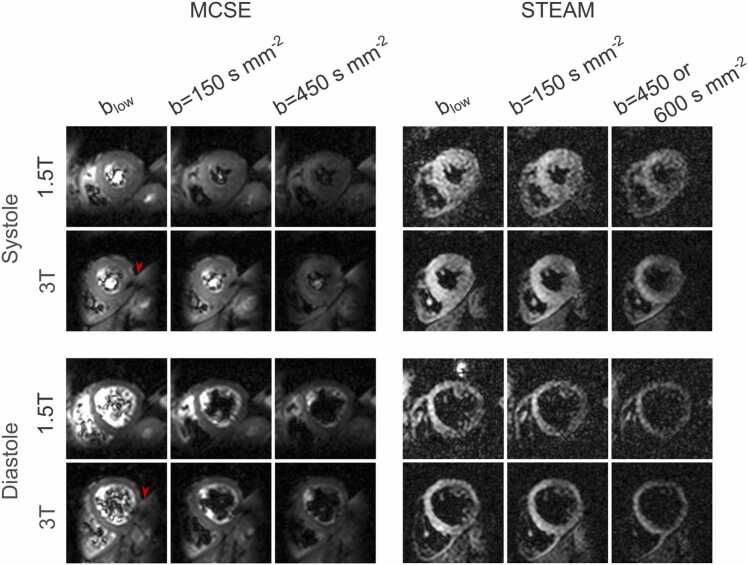
Diffusion-weighted images showing an apparent loss of SNR at 1.5T using STEAM, but not so noticeably using MCSE. Images are shown from one typical subject, acquired in both cardiac phases at both field strengths and using both sequences. Images are shown for the same diffusion encoding direction (apart from the b_low_ reference data) with the same contrast and brightness for each dataset (sequence, phase, and field strength). The red arrow heads highlight residual fat signal in the MCSE data at 3T which partially covers the LV myocardium. For equivalent full field-of-view images, see Supplementary Figs. 1 and 2. For equivalent images for all encoding directions after registration and averaging, see Supplementary Fig. 3. *SNR* signal-to-noise ratio, *STEAM* stimulated echo acquisition mode, *MCSE* motion-compensated spin-echo, *LV* left ventricle

Twenty subjects completed the scan at both field strengths (three more subjects completed only one scan due to scanner or subject availability and were excluded from further analysis) and summary demographics are shown in Table 1. Typical acquisition duration for the 10 breath holds required for each sequence and cardiac phase was around 12 min. Based on our prior experience acquiring MCSE data in diastole, these acquisitions were abandoned if the initial (2 or 3) breath holds demonstrated substantial motion-related signal loss (9 scans at 1.5T, 8 scans at 3T). Of the remaining scans, an additional 10 scored 0 in the subjective HA map assessment (0 for STEAM systole, 2 MCSE diastole 1.5T, 4 MCSE diastole 3T, and 1 for each of the other scans) and were therefore excluded from further analysis. Visually, there was an apparent loss of SNR in the diffusion-weighted images acquired using STEAM at 1.5T compared to 3T (Fig. 1 and Supplementary Figs. 1-3), but not using MCSE. However, in MCSE data acquired in some subjects (e.g. in Fig. 1), there was some unsuppressed epicardial or abdominal fat signal that was shifted to lie over part of the myocardium. The number of rejected frames (based on manual inspection) was not significantly different between field strengths for any of the acquisitions (p > 0.05 in all, see Supplementary Fig. 4) with median values ranging between 0.75 [1.65]% (median [interquartile range]) for STEAM systole at 1.5T and 4.8 [8.5]% for diastolic MCSE at 1.5T.

**Table 1:** Subject demographics.

	Mean (SD) or absolute proportion [%]
Female	11 [55%]
Male	9 [45%]
Age (years)	26 (13.5)
BMI (kg m^−2^)	23 (3.5)

*SD* standard deviation*, BMI* body mass index*.*

**Fig. 2:**
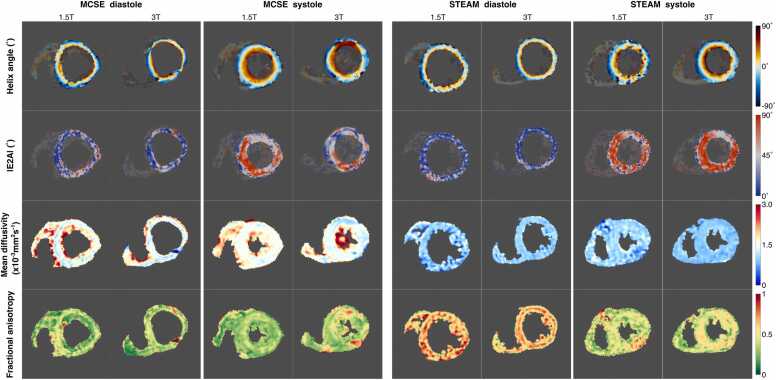
Example DT-CMR parameter maps acquired in a typical subject at all combinations of field strength, sequence type, and cardiac phase. *DT-CMR* diffusion tensor cardiovascular magnetic resonance, *STEAM* stimulated echo acquisition mode, *MCSE* motion-compensated spin-echo, *E2A* absolute value of the angle of the second eigenvector.

**Fig. 3:**
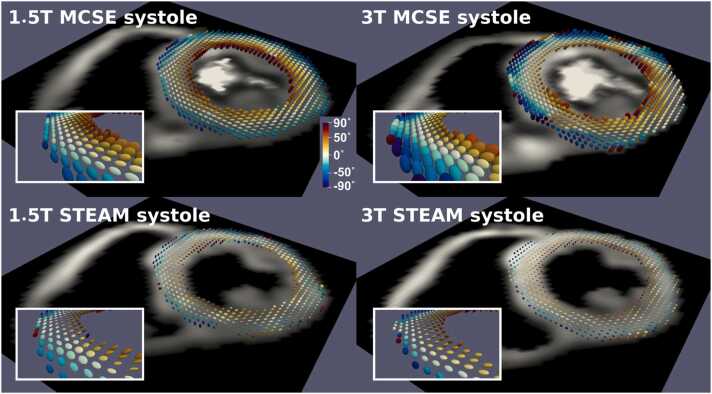
An example of the diffusion tensor represented as an ellipsoidal glyph in the LV for all systolic acquisitions in one volunteer. The glyphs are color-coded by HA. All datasets show good-quality data. Insets show a zoomed-in region from the septum. See Supplementary Fig. 5 for the equivalent data shown in diastole. *LV* left ventricle, *HA* helix angle, *STEAM* stimulated echo acquisition mode, *MCSE* motion-compensated spin-echo.

**Fig. 4:**
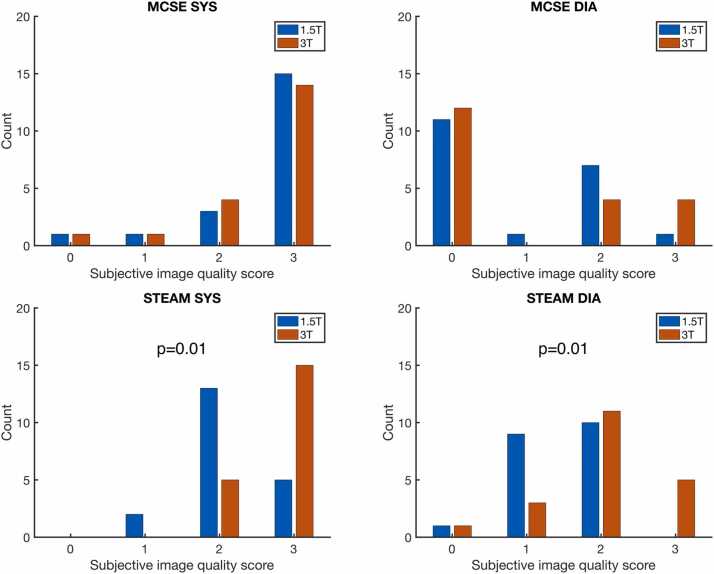
Histograms of subjective HA map quality score, showing the significant increase in the HA map appearing as expected in the 3T vs 1.5T data for STEAM but not for MCSE. Datasets scored 0 (<50% of the myocardium demonstrating the normal transmural variation in HA) were excluded from further analysis. “Counts” are equivalent to subjects. *HA* helix angle, *STEAM* stimulated echo acquisition mode, *MCSE* motion-compensated spin-echo.

DT-CMR maps appeared as expected (Fig. 2, with another example presented as ellipsoidal glyphs in Fig. 3 and Supplementary Fig. 5, with median subjective HA quality score of 3 for systolic acquisitions at 3T and systolic MCSE acquisitions at 1.5T; 1.5 for diastolic STEAM acquisitions at 1.5T; and 2 for all other combinations of cardiac phase, sequence, and field strength (Fig. 4). Subjective image quality score was improved between 1.5T and 3T data for both cardiac phases using STEAM (median 3 vs 2 in systole and 2 vs 1.5 in diastole, p = 0.01 in both), while it was not significantly different for MCSE between field strengths.

**Fig. 5:**
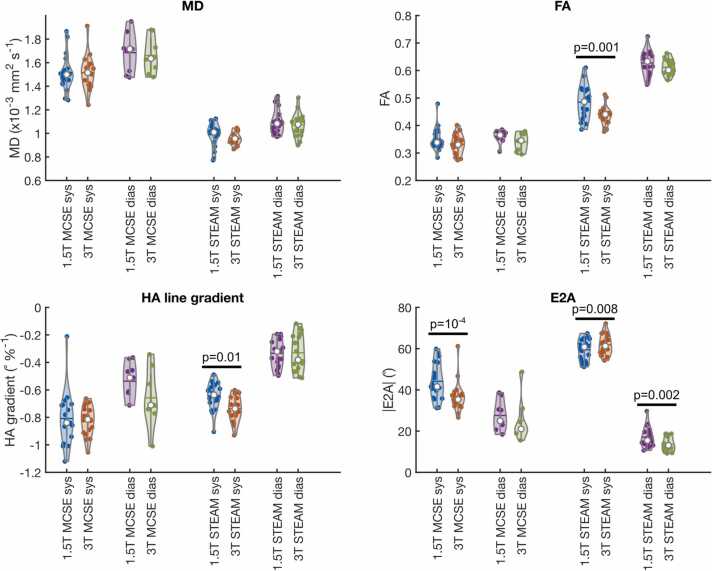
Violin plots of MD, FA, transmural HA gradient, and absolute E2A calculated without b_low_ reference data. The equivalent figure with data processed with all b-values (including b_low_ reference data are shown in Supplementary Fig. 7). The median is shown as a white circle in each plot and the mean is shown as a horizontal line. *MD* mean diffusivity, *FA* fractional anisotropy, *HA* helix angle, *E2A* absolute value of the angle of the second eigenvector, *STEAM* stimulated echo acquisition mode, *MCSE* motion-compensated spin-echo.

We compare DT-CMR parameters between field strengths from the analysis performed without b_low_ reference data in the main body and provide the equivalent results with the b_low_ images included in the tensor calculation in the Supplementary Material (see Supplementary Tables 2 and 3 for results without b_low_ and with b_low_, respectively). There was no significant difference in MD between field strength for either sequence at either cardiac phase (p > 0.05 for all, Fig. 5). However, in some subjects, there was a marked difference in the residual blood signal in the MCSE data with b = 150 smm^−2^ between field strengths. This resulted in some large variations in MD between field strength when data were analyzed without b_low_ (see Supplementary Fig. 6), although, as evidenced by the lack of a significant difference in MD between field strengths when b_low_ was excluded, this was not a systematic difference between field strengths.

For FA, there was a significant reduction in FA when going from 1.5T to 3T for STEAM in systole (0.485 ± 0.060 vs 0.435 ± 0.034, p = 0.001), while the diastolic data and systolic data for MCSE were not significantly different (p > 0.05).

The transmural gradient in HA (HA line gradient) was less steep when measured at 1.5T than at 3T with STEAM in systole (−0.644 ± 0.097° %^−1^ vs. −0.735 ± 0.091° %^−1^, p = 0.01), but not significantly different between field strengths using MCSE in either cardiac phase or using STEAM in diastole. Absolute E2A was significantly higher at 1.5T than at 3T when measured from MCSE data in systole (44.1 [8.8]° vs 36.8 [7.4]°, p ∼ 10^−4^) and using STEAM in diastole (17.0 [4.8]° vs 13.7 [3.5]°, p = 0.002), but lower at 1.5T using STEAM in systole (59.4 ± 5.2° vs 61.8 ± 4.8°, p = 0.008).

**Fig. 6:**
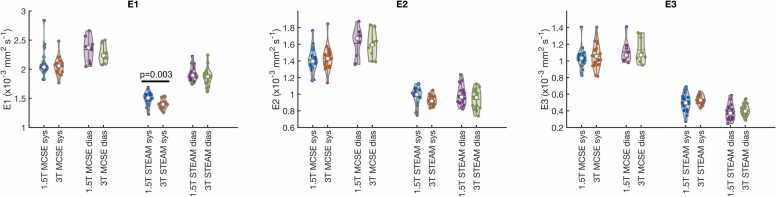
Violin plots comparing eigenvalues calculated without b_low_ reference data between field strengths. Equivalent plots calculated with the b_low_ reference data are shown in Supplementary Fig. 8. *STEAM* stimulated echo acquisition mode, *MCSE* motion-compensated spin-echo, *sys* systole, *dias* diastole.

There were no significant differences between field strengths in the eigenvalues, apart from for STEAM systole, where the first eigenvalue is higher at 1.5T than 3T (1.49 ± 0.12 × 10^−3^ mm^2^s^−1^ vs 1.409 ± 0.083 × 10^−3^ mm^2^s^−1^, p = 0.003, see Fig. 6), causing the observed increase in FA for this dataset at 1.5T over 3T.

**Fig. 7:**
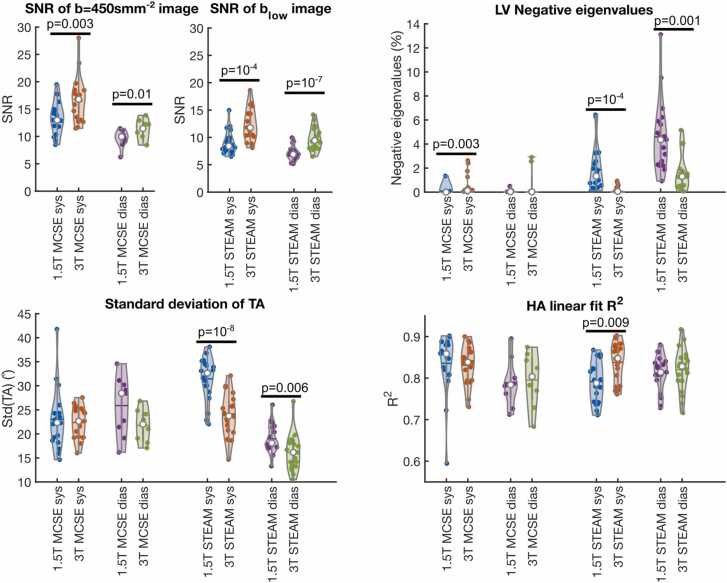
Quality metrics for data excluding the b_low_ reference apart from the SNR in the b_low_ images. See Fig. 5 for an explanation of the violin plots. Equivalent plots, processed including the b_low_ reference data, are shown in Supplementary Fig. 9 (apart from SNR). SNR values are shown for the b = 450 smm^−2^ data for MCSE data and for the b_low_ data for STEAM due to the differences in main b-value between the field strengths for STEAM and the high-intensity blood signal in the b_low_ images for MCSE. *SNR* signal-to-noise ratio, *MCSE* motion-compensated spin-echo, *STEAM* stimulated echo acquisition mode, *LV* left ventricle, *TA* transverse angle, *HA* helix angle, *sys* systole, *dias* diastole.

As expected, SNR is significantly increased at 3T over the 1.5T acquisitions for all combinations of cardiac phase and sequence (13.0 [3.1] vs 16.8 [5.2] MCSE systole, 9.6 ± 1.5 vs 11.1 ± 2.0 MCSE diastole for b = 450 smm^−2^ and 8.4 [2.4] vs 11.8 [4.0] STEAM systole, 7.1 ± 1.3 vs 9.8 ± 2.1 STEAM diastole for b_low_ data, all p < 0.05, see Fig. 7). The average ratio of SNR at 3T to the equivalent value at 1.5T is 1.3 and 1.2 for MCSE data in systole and diastole, respectively, compared to a theoretical ratio of 2.8 (see Supplementary Material). The equivalent ratios for the STEAM data were 1.4 at both cardiac phases compared to a theoretical value of 2.4. Negative eigenvalues are unphysical and are suggestive of poor-quality data. Unexpectedly, the percentage of pixels containing one or more negative eigenvalues in the LV is significantly higher for the 3T MCSE systole data than for the 1.5T data (0.09 [0.31]% vs 0.50 [0.85], p = 0.003, Fig. 7). In contrast, the proportion of pixels with negative eigenvalues is higher for 1.5T than for 3T for both STEAM acquisitions (1.9 [1.9] % vs 0.21 [0.31] %, p ∼ 10^−4^ systole and 4.6 [3.0] % vs 1.6 [1.5], p ∼ 10^−3^).

The TA is assumed to be relatively consistent across the myocardium and the standard deviation of the TA across the LV myocardium has been used as a marker of DT-CMR data quality, with higher values indicating poorer quality [15]. The standard deviation of the TA is significantly higher in the 1.5T data for STEAM acquisitions in both cardiac phases (31.5 ± 4.5° vs 23.8 ± 4.4°, p ∼ 10^−8^ STEAM diastole, 19.0 ± 3.0° vs 16.1 ± 3.8°, p = 0.005), but not significantly different for the MCSE acquisitions. The mean R^2^ of the transmural HA profiles was significantly higher (higher being suggestive of better quality data) for the systolic STEAM data at 3T (0.79 ± 0.05 vs 0.838 ± 0.046, p < 0.01), but not significantly different for any of the other sequences or cardiac phases. The proportion of transmural HA profiles reaching the threshold for inclusion in the quality metric (negative slope, R^2^ < 0.3) was also higher for systolic STEAM data at 3T than at 1.5T (83.9 ± 5.4% vs 91.4 ± 4.8%, p ∼ 10^−4^, Supplementary Fig. 10).

**Fig. 8:**
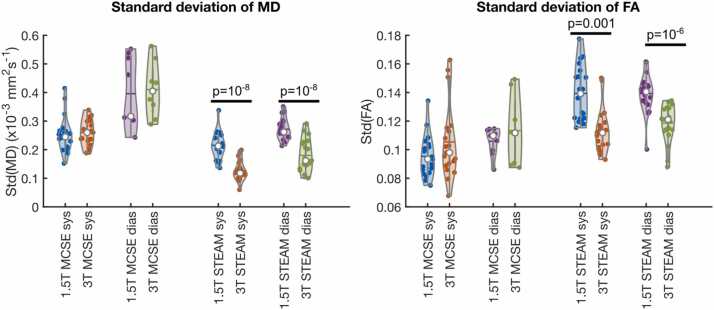
Variation of the FA and MD over the left ventricular myocardium depending on field strength from data processed without the b_low_ reference data. For an explanation of the violin plots, see Fig. 5. Equivalent plots for data with b_low_ reference data included are provided in Supplementary Fig. 11. *FA* fractional anisotropy, *MD* mean diffusivity, *MCSE* motion-compensated spin-echo, *STEAM* stimulated echo acquisition mode.

High-quality DT-CMR data in healthy volunteers would generally be associated with smooth maps of MD and FA across the LV myocardium. The standard deviation of FA and MD was found to be significantly higher for STEAM data acquired in both phases at 1.5T than at 3T (MD: 0.215 ± 0.046 × 10^−3^ mm^2^s^−1^ vs 0.127 ± 0.035 × 10^−3^ mm^2^s^−1^, p∼10^−8^ STEAM systole, 0.272 ± 0.038 × 10^−3^ mm^2^s^−1^ vs 0.180 ± 0.057 × 10^−3^ mm^2^s^−1^, p ∼ 10^−8^ STEAM diastole; FA: 0.139 [0.019] vs 0.113 [0.015], p ∼ 10^−3^ STEAM systole, 0.139 ± 0.013 vs 0.119 ± 0.013, p ∼ 10^−6^ STEAM diastole Fig. 8).

## 4 Discussion

In this first in-vivo comparison of DT-CMR performed at multiple B0 field strengths, we demonstrate that both STEAM and MCSE can effectively be performed in systole at 1.5T and 3T with relatively modest maximum gradient strengths of ≤60 mT/m. While our results are consistent with a reduction in image quality and the quality of DT-CMR measures obtained at 1.5T using STEAM, the differences in image quality and DT-CMR parameters were reduced when using MCSE. We attribute this loss of quality in the STEAM data to the substantial reduction in SNR that is associated with the reduction in B0 field strength. Both MCSE and STEAM data are sensitive to SNR and insufficient SNR results in systematic bias and imprecision in DT-CMR parameters 8, 27, 28. However, our results suggest that for the protocols used here, the SNR was sufficient at 1.5T to obtain MCSE data without significantly detriment to the DT-CMR parameters when compared to 3T data. In contrast, while STEAM data at 1.5T was generally of at least acceptable image quality (scored ≥1), there were significant differences between field strengths in a number of DT-CMR parameters and the majority of measures of DT-CMR data quality.

While a large majority of acquisitions were considered successful for STEAM in both cardiac phases and MCSE in systole, diastolic MCSE was unsuccessful in 55% of cases at 1.5T and 60% of cases at 3T. These results are broadly consistent with our prior findings in healthy volunteers [25] and hypertrophic cardiomyopathy patients [29] and show a similar trend to results from other groups in diastole [30].

The ability to perform DT-CMR at 1.5T potentially allows an expansion of DT-CMR research across a wider range of scanners for clinical research studies. A 2017 UK survey reported 79% of scanners were 1.5T and only 17% 3T [20].

The differences in DT-CMR parameters observed between field strengths cannot be directly attributed to the effect of field strength on the diffusion of water molecules. Unlike the type of diffusion preparation (STEAM or MCSE here), as far as we are aware there is no effect of a magnetic field on the thermally induced random walk of water molecules undergoing self-diffusion. The observed changes in DT-CMR parameters between field strengths are, therefore, attributable to ancillary effects associated with the acquisition. As we and others have shown previously 27, 28, a reduction in SNR can result in systematic increases or decreases in MD and FA due to eigenvalue repulsion or noise floor effects. At the b-values used in this work (<1000 smm^−2^) and spatial resolutions, a reduction in SNR, as observed when imaging at 1.5T rather than 3T, would be expected to result in an increase in measured FA and a reduction in systolic E2A [27], which is consistent with our findings. The effects of noise on HA line gradient have not been studied, but a reduction in its magnitude in systole, as we observed between 3T and 1.5T STEAM data (Fig. 5) would seem to be consistent with an increase in noise. Furthermore, while the same difference was not observed between field strengths for STEAM diastole, the 1.5T HA line gradient was of lower median magnitude at the lower field strength and if the tendency of HA line gradient is toward 0 as SNR decreases, this dependency on SNR may be more prominent with larger ground truth HA line gradient.

The main b-value for the STEAM data was reduced at 1.5T due to the visible loss in SNR in our initial pilot studies, while this may have contributed to the observed increase in FA at 1.5T [24] we do not believe that this change had a substantial effect on the quality of the diffusion tensor data. We based our selected b values on previous studies 24, 27, 28, showing that for a given SNR and diffusion tensor increasing the main b value increases the accuracy of the diffusion up to a point (via a reduction in eigenvalue repulsion and an increase in precision) until noise floor effects begin to dominate. There were also differences between the acquisitions due to the differing gradient performance on the two scanners used. The 1.5T scanner had a maximum gradient strength of 45 mT/m, while the 3T scanner could achieve 60 mT/m, resulting in a reduction in MCSE TE from 74 ms to 62 ms, but had almost negligible effect on the STEAM sequences. This TE reduction in the MCSE sequence will contribute to the observed increased SNR in our 3T data, but it also reduces the effective diffusion encoding time (as the encoding gradients are shorter) although not sufficiently to result in any observable changes in the measured DT-CMR parameters. The recent introduction of ultrahigh strength whole body gradient sets in commercially available scanners is likely to enable substantial reductions in TE below the values used here, which is particularly important in DT-CMR due to the short T2 and highly dynamic nature of the myocardium. These next-generation scanners are currently 3T systems, but 1.5T ultra-high gradient systems would be an interesting future prospect for in-vivo DT-CMR.

There was also a field strength dependence of the severity of the fat artifacts. The STEAM sequence provides excellent fat suppression with a combination of an initial chemical shift selective fat saturation pulse followed by rapid decay of any residual fat magnetization during the mixing time (TM) due to the short T1 of fat. However, the fat signal was more problematic in MCSE as the chemical shift selective fat suppression pulse did not fully suppress all of the fat signals. The residual fat signal was shifted in the phase encode direction by the relatively low effective bandwidth in this direction compared to the frequency encode direction in the single shot EPI readout. As the EPI readouts were matched between field strengths (giving the same echo spacing and therefore, the same phase encode bandwidth), the constant chemical shift in parts per million (ppm) field strength resulted in a shift in the location of the fat signal in the images which is double the size at 3T compared to 1.5T (3.4 ppm is 220 Hz at 1.5T and 440 Hz at 3T). While in some subjects we were able to move the shifted epicardial or liver fat signal away from the myocardium by changing the phase encode orientation, in a number of studies the fat signal at 3T resulted in artifacts in the LV myocardium of the DT-CMR results (see Supplementary Fig. 12). Careful examination of the data has shown that these fat artifacts were the cause of the significant increase in the proportion of negative eigenvalues in the 3T systolic MCSE data compared to the equivalent 1.5T data. Binomial water selective excitation pulses were not available as part of the custom sequences used in this study but could result in improvements in fat suppression in future MCSE studies at the expense of a very small increase in TE.

Measured SNR, as expected was significantly increased at 3T over 1.5T, although the ratio of the values at 3T to 1.5T was less than the theoretical values we obtained. There are a number of possible contributions to this lower than expected increase in measured SNR at 3T which include limitations with the SNR measurement technique due to residual blood signal (in MCSE), the impact of bulk motion between breath holds on the noise measures and partial volume artifacts at the edge of the myocardium; increased T2* related dephasing at 3T during the EPI readout; reductions in B1 transmit homogeneity and more severe fat artifacts in the 3T data than at 1.5T.

While no other study has compared in-vivo cardiac DT-CMR between field strengths, a recent study compared results of ex-vivo DT-CMR in explanted human hearts at 1.5T and 3T using multi-shot spin echo sequences and, consistent with our findings, found no significant differences in DT-CMR parameters between field strengths. While the first in-vivo cardiac diffusion studies were performed at 1.5T 2, 31, 32, DT-CMR has been predominantly performed at 3T in the recent past 13, 33, 34, 35, 36, although work from one group has reported a number of important technical developments and clinical research findings using STEAM [37] and MCSE at 1.5T 38, 39. Stoeck et al. [37] acquired STEAM data in systole and diastole at 1.5T, reporting values for mid-ventricular diastolic MD before strain correction (0.92 ± 0.11 × 10^−3^ mm^2^s^−1^ vs 1.15 ± 0.079 × 10^−3^ mm^2^s^−1^ in our study with b_low_) and systolic MD (0.88 ± 0.14 × 10^−3^ mm^2^s^−1^ vs 1.065 [0.093] × 10^−3^ mm^2^s^−1^ in our study) slightly lower than the values we obtained. Our FA results are similar to those reported by Stoeck et al. [37] at 1.5T (0.61 ± 0.04 vs 0.605 ± 0.023 in our study) in diastole but our results were slightly lower in systole (0.52 ± 0.05 vs 0.455 ± 0.036 in our study). While Stoeck et al. [37] use a different definition of sheetlet angle, based on the third eigenvector rather than that used here based on the second eigenvector, they do quote transmural HAGs which are higher than those we found (diastole: −0.73 ± 0.06° %^−1^ vs −0.334 ± 0.097° %^−1^ in our study; −1.00 ± 0.11° %^−1^ vs −0.644 ± 0.094° %^−1^ in systole), although there is a slight difference in the calculation method. From the same group, Gotschy et al. [38] report mid-ventricular septal mid-systolic DT-CMR results from MCSE data at 1.5T in a control group of 10 subjects with MD values 1.41 ± 0.07 × 10^−3^ mm^2^s^−1^ vs 1.51 ± 0.15 × 10^−3^ mm^2^s^−1^ at 1.5T in systole without b_low_ in our study, FA values of 0.35 ± 0.03 vs 0.338 [0.050] in our study (median and IQR as non-normally distributed), transmural HAG (− 1.02 ± 0.14 vs −0.84 [0.19]° %^−1^ in our study) and E2A 33.9 ± 9.5° vs 45 [15]°.

In a previous study, we reported DT-CMR results at 3T for STEAM and MCSE sequences acquired in healthy volunteers in systole and diastole [25] (albeit on a different scanner to the 3T system used here). Our MD, transmural HAG and E2A results from this study were all within 1 standard deviation or interquartile range of the equivalent results from our previous study (see Supporting Table S1 in [25]). While our STEAM FA values in diastole were also within 1 standard deviation or interquartile range of our previous results, the systolic STEAM FA values were marginally lower this time (0.47 [0.03] vs 0.435 ± 0.034) and the FA results from MCSE in this work are slightly lower than those we reported previously (diastole: 0.41 [0.07] vs 0.346 [0.062] here; systole: 0.40 [0.09] vs 0.330 [0.050] here), which could be a consequence of small differences in acquisition protocol and processing (e.g. TR = 1RR interval vs 2RR-intervals here; TE = 76 ms vs 62 ms here; binomial water selective excitation vs spectrally selective fat saturation here; rigid registration with manual thresholding of blood signal vs b-spline based non-rigid registration without thresholding here; linear tensor fitting vs non-linear tensor fitting here).

The results obtained with the inclusion of the lowest b-value (that we refer to as b_low_) show more significant differences in DT-CMR parameters and quality metrics between field strengths (see Supplementary Figures) than the data using b = 150 smm^−2^ as a reference. In particular, we see a significant increase in all eigenvalues at 1.5T in both cardiac phases using MCSE when all b-values are included (Supplementary Fig. 3). In a number of subjects, the mean primary eigenvalue exceeded the diffusion of free water at body temperature (3 × 10^−3^ mm^2^s^−1^) when b_low_ was included in the MCSE data, particularly for diastolic data at 1.5T. This is a consequence of unsuppressed moving blood signal at the epicardial border and the increase in datasets with non-physical primary eigenvalues perhaps represents poorer registration performance at 1.5T than at 3T due to the reduced SNR. Non-zero reference b-values have been used to reduce the effects of microvascular perfusion on measures DT-CMR parameters [24], although recent simulations suggest that perfusion still affects STEAM data and MCSE is relatively immune to perfusion effects at all b-values 40, 41. One key reason for using non-zero reference b-values in MCSE data is that the blood signal is reduced at higher b-values. Blood signal on the endocardial border contaminates the DT-CMR results, resulting in elevated MD values (due to the higher diffusion coefficient and mobile nature of blood), but removal of these pixels from the analyzed region removes endocardial pixels where there appear to be meaningful angular (HA and E2A) values. While blood signal is usually reduced at non-zero b-values, slow-flowing blood at the borders often still produces signal and partial volume effects may also mean that myocardial signal is also affected. We found inconsistent suppression of blood signal at b > b_low_ between scans within the same subject (see Supplementary Fig. 5), which resulted in substantial changes in MD between field strengths in some subjects. Despite our best efforts to match slice positioning and orientation between scans at different field strengths, there were inevitable small differences and we attribute this inconsistency in blood suppression to these minor variations in slice location/orientation. Future studies might consider using non-zero b-values for STEAM to minimize the contribution of microvascular perfusion on the calculated DT-CMR parameters. For future MCSE studies, the use of non-zero reference b-values and potentially different regions of interest for angular and rotationally invariant parameters should be considered to minimize but not eliminate the effects of slow-flowing blood at the epicardium on the DT-CMR parameters.

We adapted our processing methods from previous studies [21] to incorporate non-rigid registration, which we found to result in much improved registration for the MCSE data compared to the rigid registration that we have reliably used in the past for STEAM. One important reason for the use of non-rigid registration is the eddy current-related distortions of the MCSE data, which are much less severe in the STEAM data due to the much smaller diffusion encoding gradients required.

### 4.1 Limitations

While we were careful to match acquisition duration and spatial resolution between field strengths and sequences, we did use more averages, fewer slices, and lower spatial resolution than is typical in many MCSE studies 16, 36, 38. It may be that the SNR efficiency of the MCSE sequences would permit a reduction in the number of averages acquired or an increase in the spatial resolution without substantial detrimental effect, particularly at 3T. While alternative MCSE protocols may have resulted in different conclusions, we chose to base our resolution and field of view on one that has proved to be robust in a number of clinical studies, resulting in new insights into the microstructural origins of a variety of cardiac pathologies 3, 18, 34, 42.

We optimized the phase encode direction for each individual acquisition based on the visually apparent SNR and magnitude and location of residual fat artifacts. This resulted in differences between field strength, sequence, and cardiac phase and therefore variations in the location and extent of off-resonance-related distortion. However, the short EPI readout means these distortions were relatively small and any differences would be distributed between the field strengths.

Our cohort was on average relatively young with a healthy body mass index, although it spanned a range of ages between 20 and 63 years and habitus between underweight and obese. However, based on our previous experience with these sequences 25, 29, we would expect our results to generalize to many patient populations.

## 5 Conclusion

While distances diffused are independent of field strength, the SNR and difference in image artifacts between field strengths can affect the DT-CMR parameters measured. Both MCSE and STEAM are effective at 1.5T and 3T in systole and STEAM is also effective in diastasis. While the data quality and DT-CMR parameters obtained using MCSE were minimally affected by field strength, the quality of data obtained using STEAM benefits from the SNR available at 3T. DT-CMR studies could consider making use of 1.5T hardware where access to 3T scanners is more difficult particularly where MCSE sequences are to be used with systolic triggering.

## Funding

This work was funded by 10.13039/501100000274British Heart Foundation grant RG/19/1/34160. Andrew D. Scott would like to acknowledge funding from Engineering and Physical Sciences Research Council Grant EP/X014010/1. Ke Wen and Yaqing Luo would like to acknowledge funding from the EPSRC Centre for Doctoral Training in Smart Medical Imaging (EP/S022104/1). Jiahao Huang is partly funded by the UKRI Future Leaders Fellowship (MR/V023799/1).

## Author contributions

**Andrew David Scott:** Writing – review and editing, Writing – original draft, Supervision, Software, Methodology, Investigation, Funding acquisition, Formal analysis, Data curation, Conceptualization. **Sonia Nielles-Vallespin:** Writing – review and editing, Supervision, Methodology, Conceptualization. **Dudley J. Pennell:** Writing – review and editing, Supervision, Resources, Funding acquisition, Conceptualization. **Pedro F. Ferreira:** Writing – review and editing, Visualization, Software, Conceptualization. **Rajkumar Soundarajan:** Writing – review and editing, Methodology, Investigation. **Simon Gover:** Writing – review and editing, Methodology, Investigation. **Jiahao Huang:** Writing – review and editing, Investigation. **Yaqing Luo:** Writing – review and editing, Investigation, Data curation. **Ke Wen:** Writing – review and editing, Investigation, Formal analysis, Data curation.

## Declaration of competing interests

The authors declare the following financial interests/personal relationships which may be considered as potential competing interests: The Cardiovascular Magnetic Resonance Unit at the Royal Brompton Hospital receives research support from Siemens. Ke Wen and Yaqing Luo are partly funded by Siemens.
