# Supplementary material for: The effects of field strength on stimulated echo and motion-compensated spin-echo diffusion tensor cardiovascular magnetic resonance sequences
Source: J Cardiovasc Magn Reson. 2024 Jun 25;26(2):101052. doi: 10.1016/j.jocmr.2024.101052 (PMC11283220; doi:10.1016/j.jocmr.2024.101052)
Supplement: Supplementary file 1 — Supplementary material [file mmc1.pdf]

Supplementary material

SNR at 1.5T and 3T for MCSE and STEAM sequences

We previously described the theoretical ratio of signal to noise values between MCSE and STEAM acquisitions matched for spatial resolution[1]. Here we adapt the same theory to show the theoretical ratio between acquisitions performed at 3T and 1.5T using either STEAM or MCSE.

Assuming that the diffusivity is identical between field strengths, for STEAM:

$$\frac{SNR_{STEAM}(3T)}{SNR_{STEAM}(1.5T)} = \frac{2\cdot\left(1-e^{\frac{-Trecov_{STEAM}}{T1(3T)}}\right)\cdot e^{\frac{-TE_{STEAM}(3T)}{T2(3T)}}\cdot e^{\frac{-TM}{T1(3T)}}}{\left(1-e^{\frac{-Trecov_{STEAM}}{T1(1.5T)}}\right)\cdot e^{\frac{-TE_{STEAM}(1.5T)}{T2(1.5T)}}\cdot e^{\frac{-TM}{T1(1.5T)}}} \quad \text{(supplementary equation 1)}$$

and for MCSE:

$$\frac{SNR_{MCSE}(3T)}{SNR_{MCSE}(1.5T)} = \frac{2\cdot\left(1-e^{\frac{-Trecov_{MCSE}}{T1(3T)}}\right)\cdot e^{\frac{-TE_{MCSE}(3T)}{T2(3T)}}}{\left(1-e^{\frac{-Trecov_{MCSE}}{T1(1.5T)}}\right)\cdot e^{\frac{-TE_{MCSE}(1.5T)}{T2(1.5T)}}} \quad \text{(supplementary equation 2)}$$

where $Trecov_{STEAM}$ and $Trecov_{MCSE}$ are the time allowed for T1 recovery between repeats of the sequence and equal to 1 RR interval and 2 RR intervals respectively (due to the equal TRs between sequences, but the additional RF pulse in the second heartbeat of every stimulated echo shortening the recovery time). $TE_{MCSE}(FS)$ and $TE_{STEAM}(FS)$ are the echo times for the MCSE and STEAM sequences for acquisitions at the field strength FS. TM is the mixing time over which the magnetisation that contributes to the stimulated echo is stored during the STEAM sequence and is equal to 1 RR interval. The factor of 2 in the numerator accounts for the increase in difference between the two spin populations due to the increase in field strength.

Supplementary table 1 below provides values for each of these parameters from the sequences used in the study assuming a heart rate of 60 beats per minute or from the literature in the case of T1 and T2 at the two field strengths [2].

Supplementary table 1

Parameter	1.5T MCSE	3T MCSE	1.5T STEAM	3T STEAM
$Trecov$ (ms)	2000	2000	1000	1000
TE (ms)	74	64	26	25
$T1$ (ms)	1030	1471	1030	1471
$T2$ (ms)	1030	1471	40	47
$\frac{SNR(3T)}{SNR(1.5T)}$	2.8		2.4	

If we separate the contributions of the SNR ratio into spin populations (factor of 2), T2 (TE) and T1 ($Trecov$), we find that for MCSE and STEAM the ratio attributable to differences in T1 is 1.06 and 0.86. This means that the incomplete T1 recovery between repetitions of the MCSE sequence results in a reduction in relative signal available for imaging at 3T compared to 1.5T, while for the STEAM sequence the increased proportion of magnetisation stored during TM outweighs the signal loss due to reduced recovery of the longitudinal signal between sequence repeats at 3T compared to 1.5T. The SNR ratios attributable to differences in T2 and TE are 1.63 and 1.13 for MCSE and STEAM due to the substantial reduction in TE at 3T using MCSE and the shorter T2 at 1.5T.

References

1. Scott AD, Nielles-Vallespin S, Ferreira PF, Khalique Z, Gatehouse PD, Kilner P, et al. An in-vivo comparison of stimulated-echo and motion compensated spin-echo sequences for 3 T diffusion tensor cardiovascular magnetic resonance at multiple cardiac phases. J Cardiovasc Magn Reson. 2018;20.
2. Stanisz GJ, Odrobina EE, Pun J, Escaravage M, Graham SJ, Bronskill MJ, et al. T 1 , T 2 Relaxation and Magnetization Transfer in Tissue at 3T. 2005;512:507–12.

Supplementary figures

Supplementary figure 1: Full field of view images for the MCSE sequence, equivalent to the cropped images shown in figure 1.

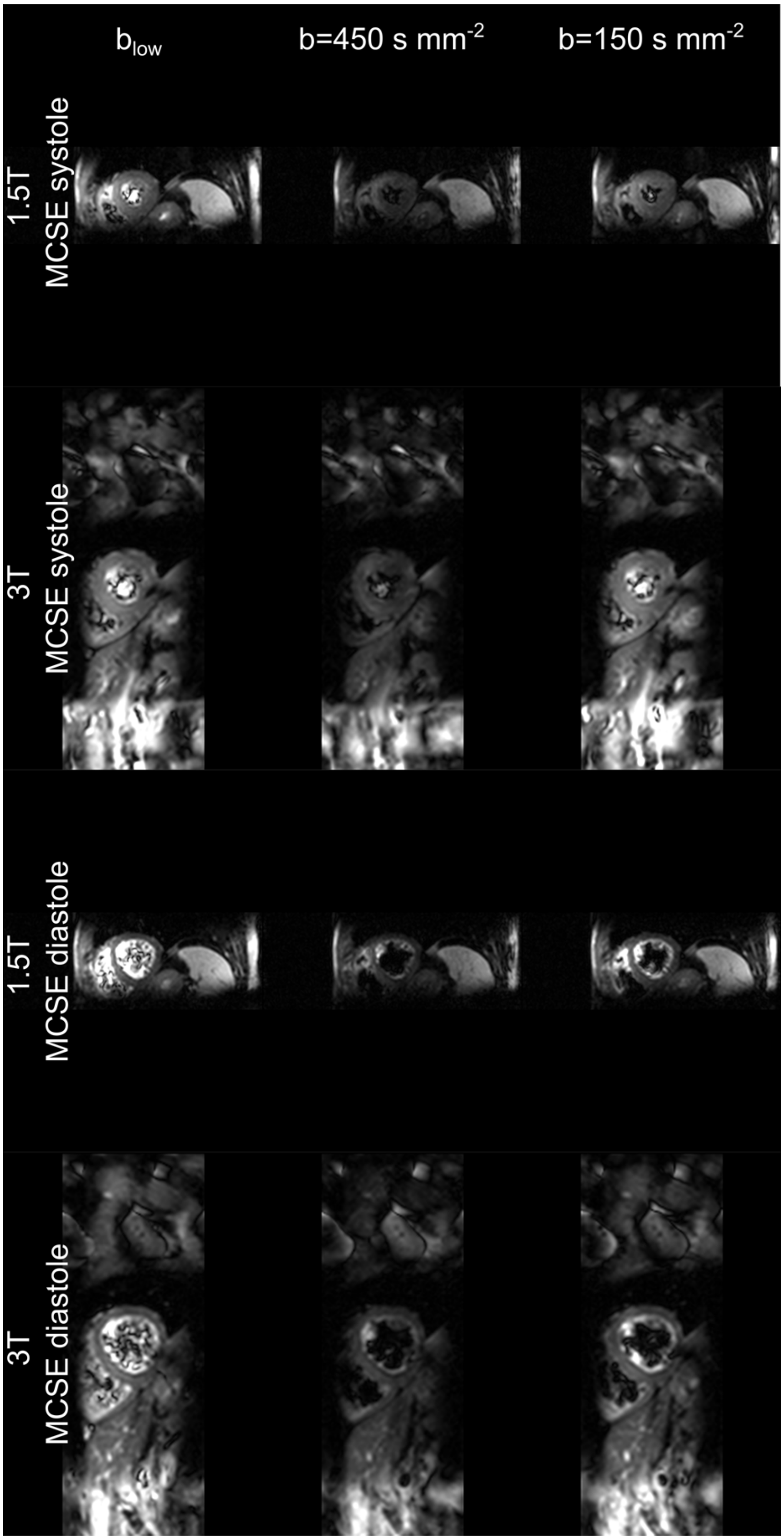

Supplementary figure 2: Full field of view images for the STEAM sequence, equivalent to the cropped images shown in figure 1.

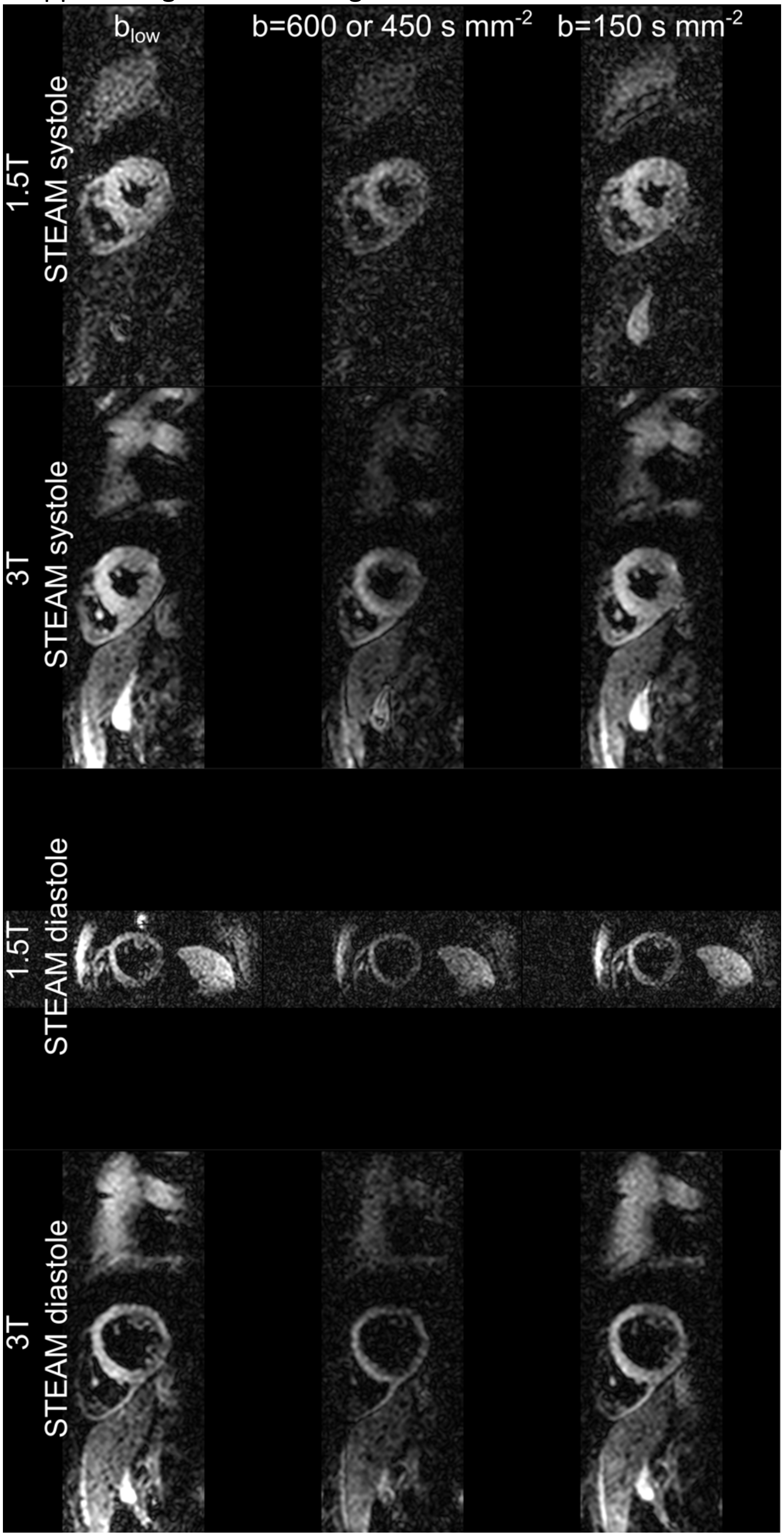

Supplementary figure 3: Registered and averaged and cropped images for both field strengths, sequences and cardiac phases. Images are shown for all b-values and diffusion encoding directions acquired.

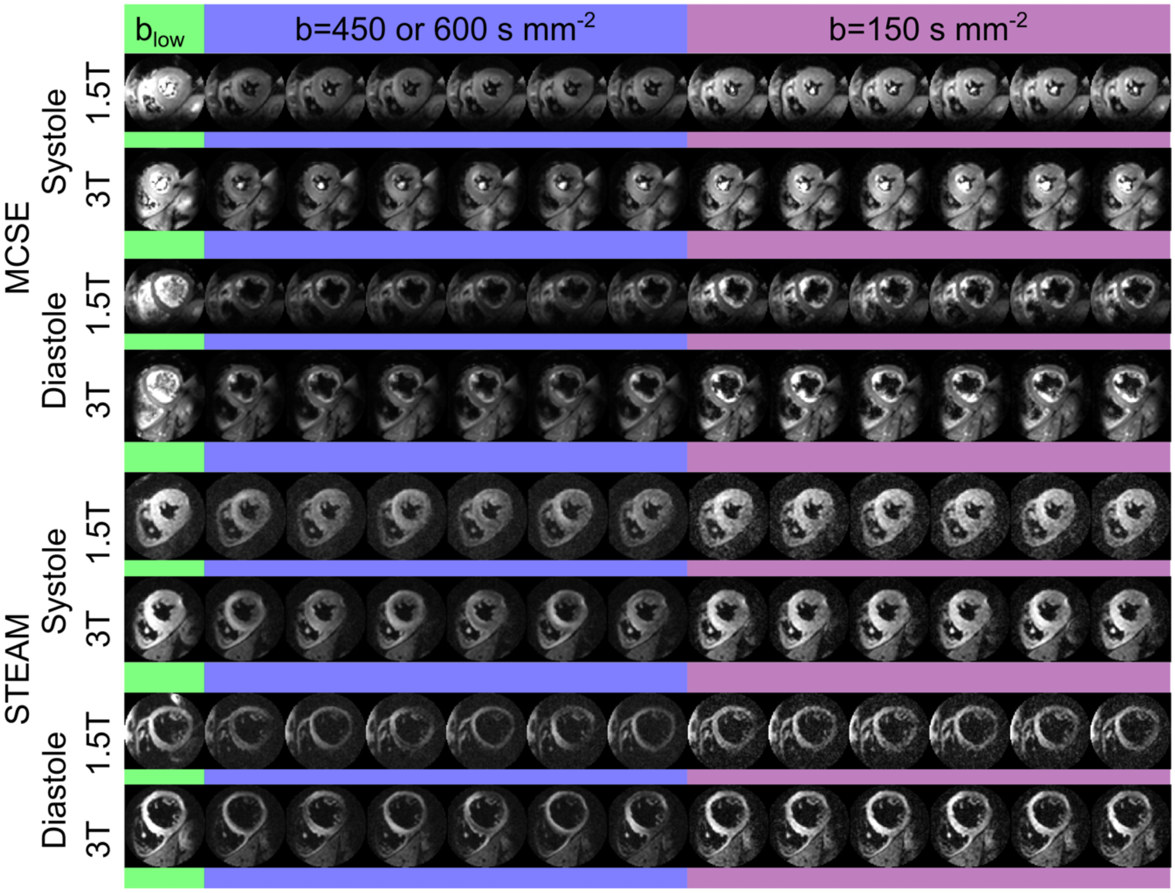

Supplementary figure 4: Violin plots comparing the percentage of frames that were rejected based on manual inspection between equivalent acquisitions at 1.5T and 3T. There were no significant differences in any of these comparisons.

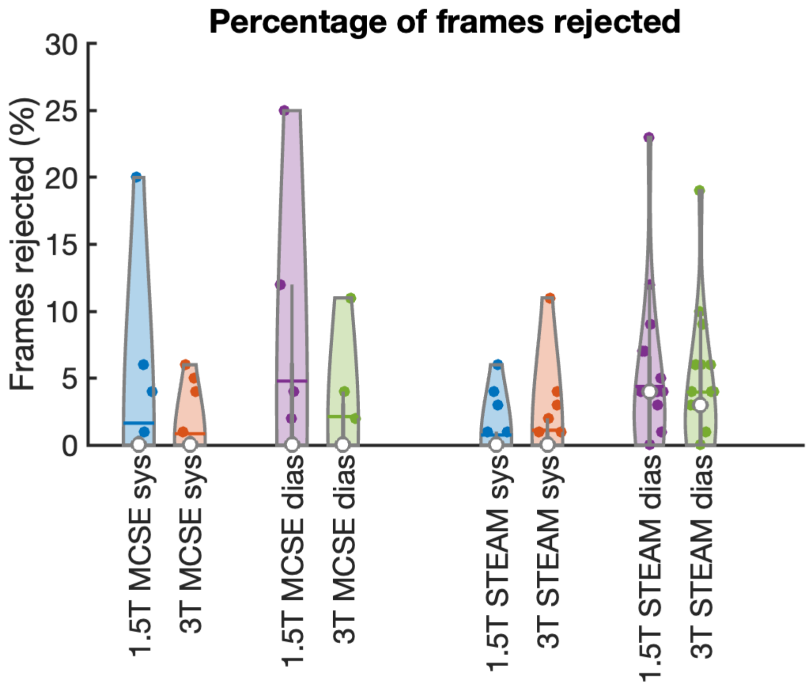

Supplementary figure 5: Superquadric ellipsoidal glyphs for the same subject shown in figure 3, but acquired in diastole. Note that in this subject the diastolic MCSE data at 3T was not scored 0 in the subjective assessment and was therefore removed from further analysis and is not shown here.

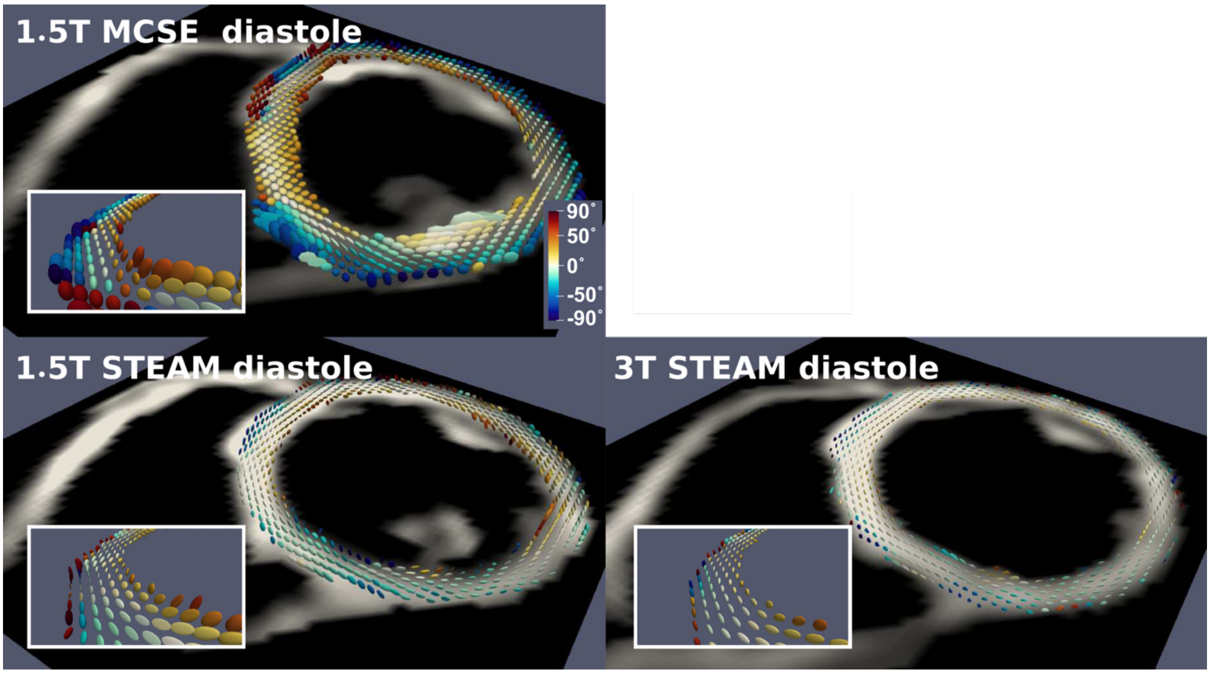

Supplementary figure 6: The intensity of the blood signal within the ventricles in the MCSE acquisitions varied between scans in the same subjects. In subject shown in the example here, at 1.5T the majority of blood signal was well supressed at b=150smm^{-2}, while at 3T, there is considerable residual blood signal at the same b-value. As a consequence, there is much higher MD in the 3T data compared to the 1.5T data shown in this example. This was not a systematic difference between field strengths and we attribute this to small variations in slice positioning and orientation between the two scans.

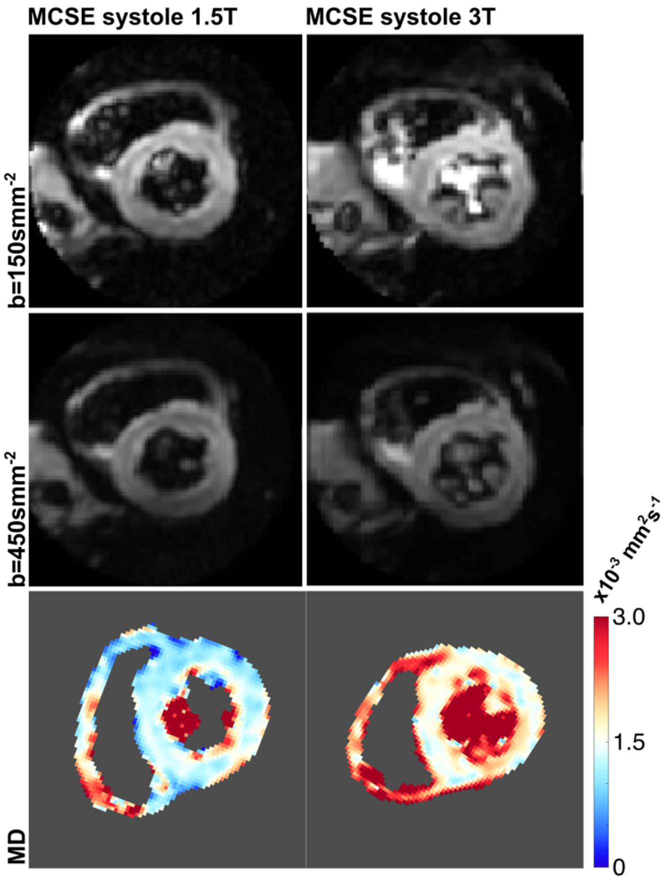

Supplementary figure 7: Violin plots comparing MD, FA, transmural HA gradient and absolute second eigenvector orientation (E2A) between field strengths with the b_{low} reference data included in the analysed data. Equivalent to figure 5 in the main manuscript.

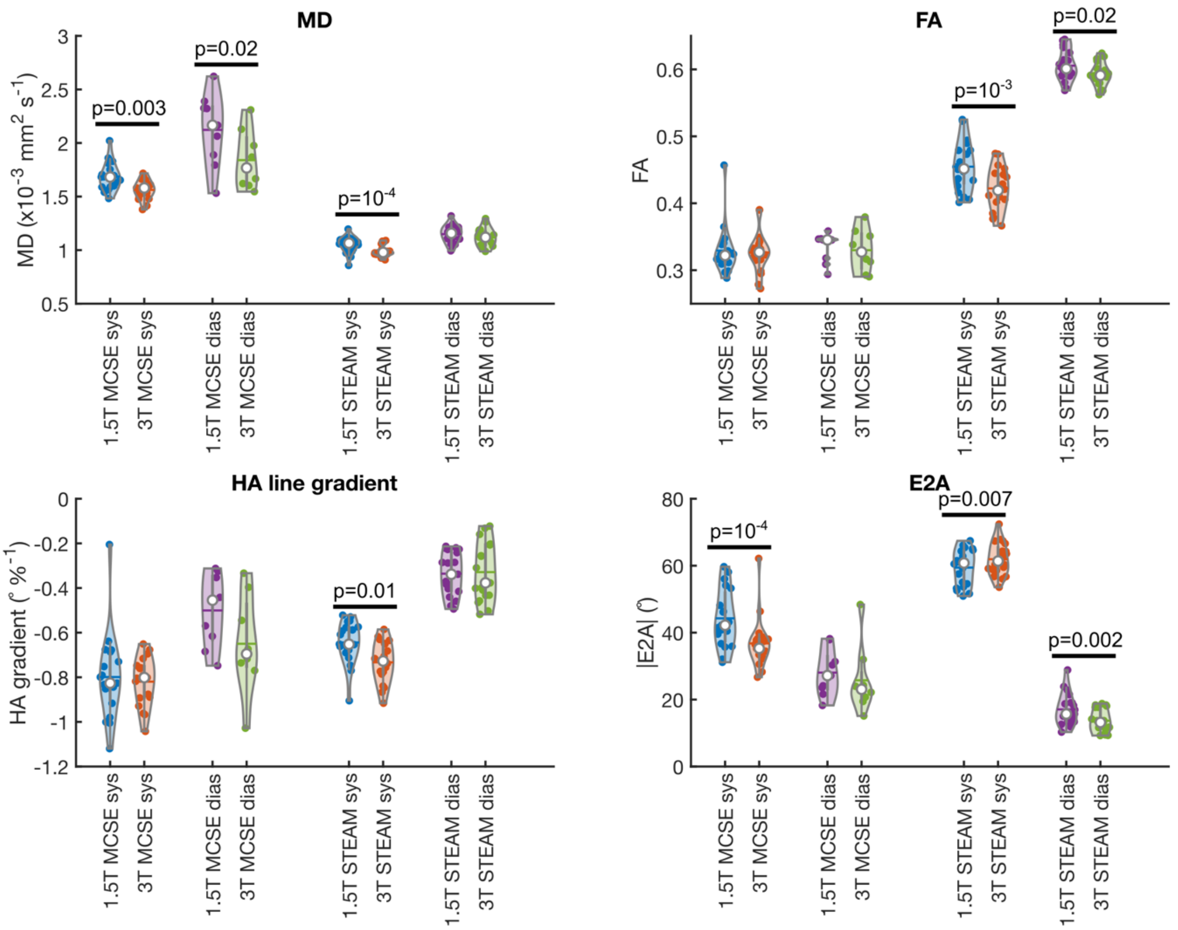

Supplementary figure 8: Violin plots comparing the diffusion tensor eigenvalues between field strengths calculated with reference b_{low} data included. See figure 6 in the main manuscript for the equivalent plots without b_{low} reference included in the analysis.

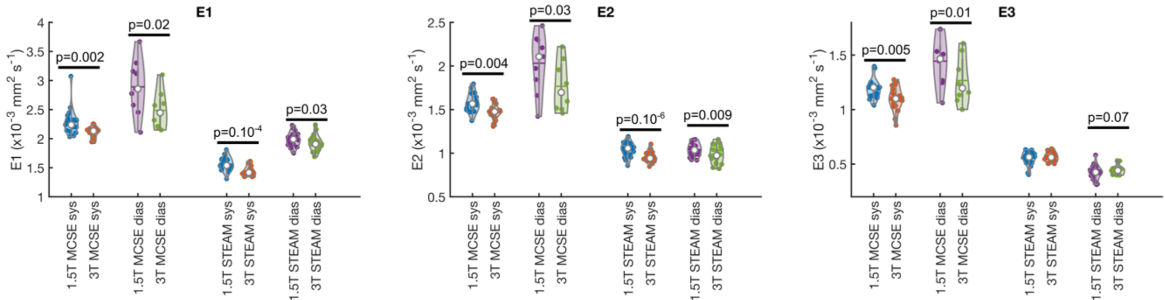

Supplementary figure 9: Violin plots showing DT-CMR data quality metrics calculated with b_{low} reference data included comparing between field strengths. Equivalent plots for data without b_{low} reference data are shown in figure 7 in the main manuscript.

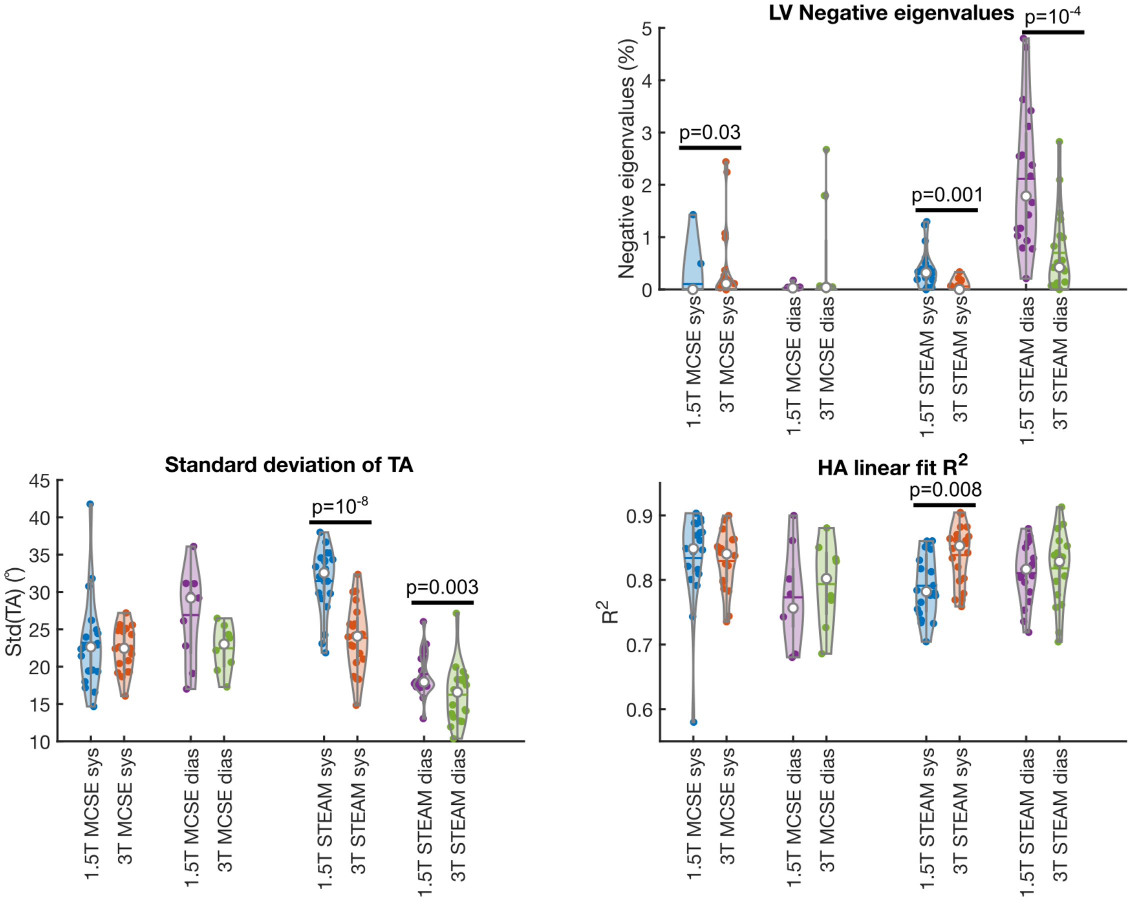

Supplementary figure 10: The proportion of HA line profiles meeting the minimum quality criteria (R^2>0.3) for analyses both with and without b_{low} reference data included in the analysis.

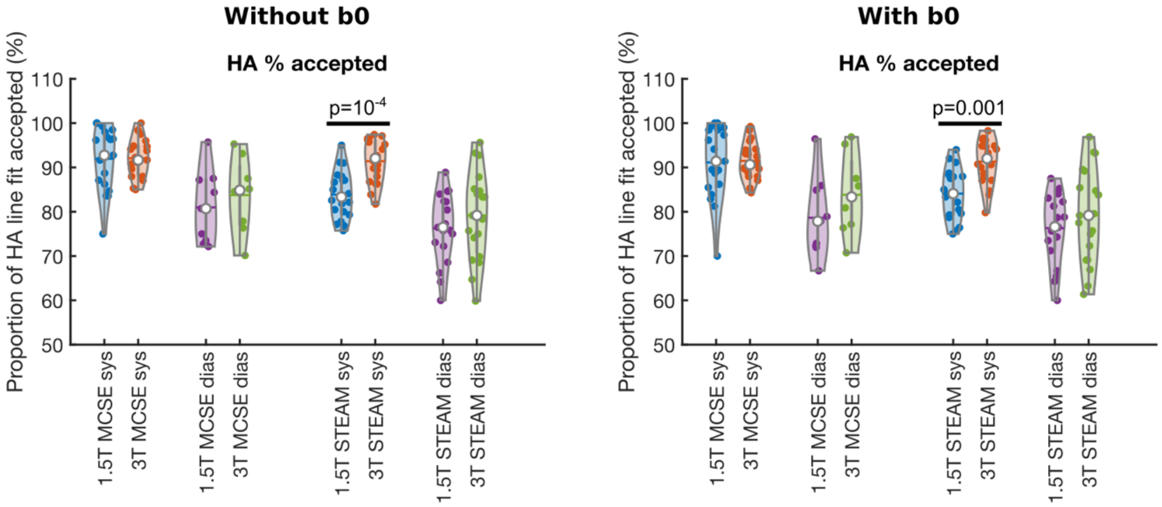

Supplementary figure 11: Violin plots comparing the standard deviation of the MD and FA over the myocardium between 1.5T and 3T for the data analysed with b_{low} reference data included. For equivalent plots for the data without b_{low} reference data, see figure 8 in the main manuscript.

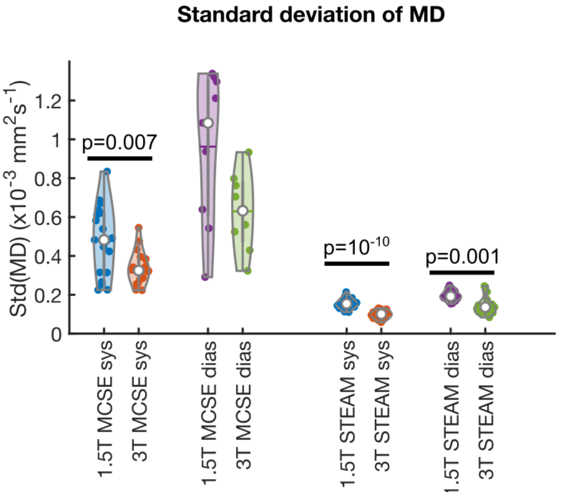

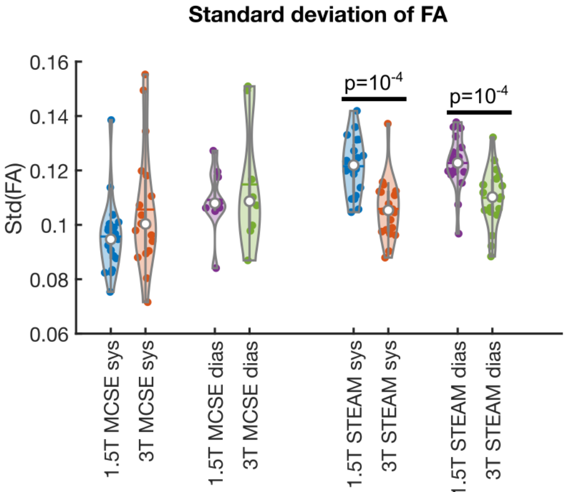

Supplementary figure 12: Unsuppressed fat artefacts in MCSE data at 3T. In the upper row the epicardial fat in the atrioventricular groove is shifted posteriorly (towards the right in the image) along the phase encode direction in the MCSE image, impinging on the LV myocardium. In the lower row, epicardial fat surrounding the LV free wall is shifted anteriorly (left in the image) along the phase encode direction, impinging on the LV myocardium. Measurements superimposed on the images show the fat shift distance based on the echo spacing, parallel imaging factor and fat chemical shift at 3T.

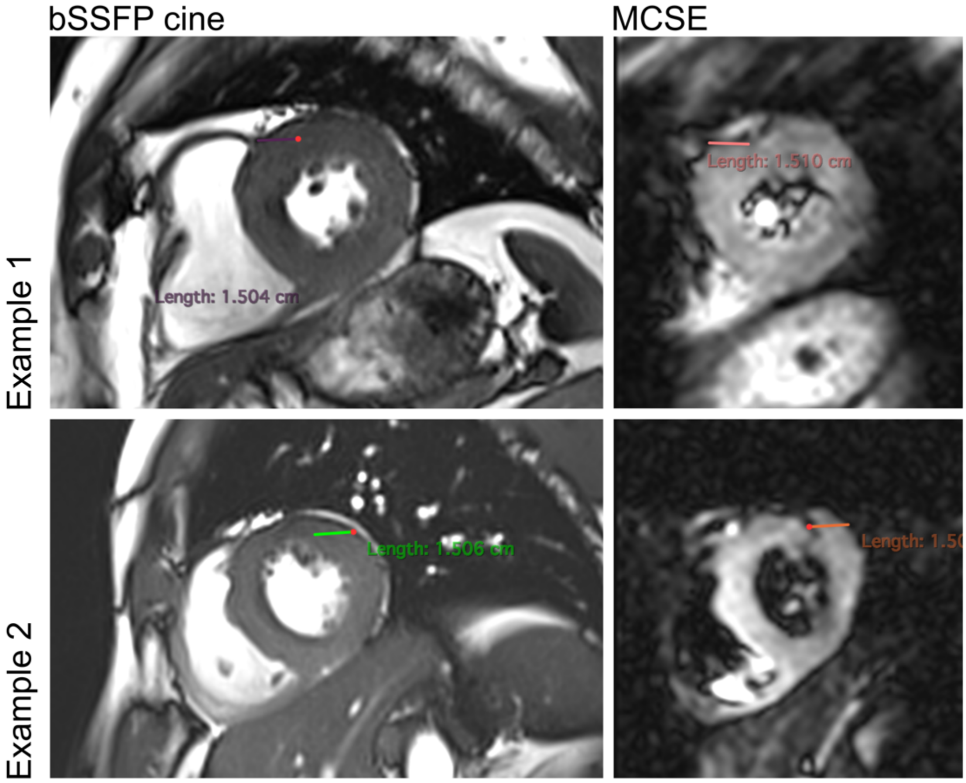

Additional supplementary tables

Supplementary table 2: A summary of DT-CMR parameters at 1.5T and 3T calculated from the data without b_{low} reference data. Where a Shapiro-Wilks test for normality has p<0.05 at either of the field strengths in a comparison, the data is shown as median [interquartile range] and as mean±standard deviation in other cases. Comparisons where a pairwise (t-test for normally distributed data and a Wilcoxon sign-rank test otherwise) test demonstrated a significant difference between field strengths are indicated by *.

Parameter	MCSE Systole 1.5T	MCSE Systole 3T	MCSE Diastole 1.5T	MCSE Diastole 3T	STEAM Systole 1.5T	STEAM Systole 3T	STEAM Diastole 1.5T	STEAM Diastole 3T
MD ($x10^{-3}$ mm^2s^{-1})	1.51±0.15	1.51±0.14	1.69±0.16	1.66±0.16	0.99±0.10	0.957±0.050	1.09 [0.13]	1.08 [0.11]
FA	0.338 [0.050]	0.330 [0.050]	0.365 [0.025]	0.346 [0.062]	0.485±0.060*	0.435±0.034*	0.627±0.043	0.612±0.026
HALG ($^\circ$ $\%^{-1}$)	-0.84 [0.21]	-0.81 [0.16]	-0.54±0.13	-0.66±0.21	-0.644±0.097*	-0.735±0.091*	-0.33±0.10	-0.33±0.13
|E2A| (°)	45 [15]*	35.4 [5.5]*	25.0 [9.8]	21.0 [7.8]	59.4±5.2*	61.8±4.8*	15.5 [5.9]*	13.1 [6.5]*
E1 ($x10^{-3}$ mm^2s^{-1})	2.04 [0.12]	2.06 [0.14]	2.34±0.21	2.26±0.15	1.49±0.12*	1.409±0.083*	1.93±0.12	1.87±0.15
E2 ($x10^{-3}$ mm^2s^{-1})	1.40 [0.13]	1.43 [0.15]	1.61±0.18	1.59±0.17	0.98±0.11	0.928±0.056	0.99±0.12	0.94±0.11
E3 ($x10^{-3}$ mm^2s^{-1})	1.03 [0.10]	1.05 [0.19]	1.07 [0.15]	1.07 [0.29]	0.49±0.10	0.535±0.050	0.39±0.10	0.407±0.063

Supplementary table 3: A summary of DT-CMR parameters at 1.5T and 3T calculated from the data with b_{low} reference data included in the analysis. Where a Shapiro-Wilks test for normality has $p<0.05$ at either of the field strengths in a comparison, the data is shown as median [interquartile range] and as mean±standard deviation in other cases. Comparisons where a pairwise (t-test for normally distributed data and a Wilcoxon sign-rank test otherwise) test demonstrated a significant difference between field strengths are indicated by *.

Parameter	MCSE				STEAM			
	Systole		Diastole		Systole		Diastole	
	1.5T	3T	1.5T	3T	1.5T	3T	1.5T	3T
MD ($x10^{-3}$ mm^2s^{-1})	1.69±0.13*	1.562±0.088*	2.12±0.34*	1.84±0.28*	1.065 [0.093]*	0.979 [0.038]*	1.149±0.079	1.119±0.083
FA	0.322 [0.029]	0.327 [0.022]	0.345 [0.031]	0.327 [0.052]	0.455±0.036*	0.422±0.031*	0.605±0.023*	0.592±0.017*
HALG (° $\%^{-1}$)	-0.83 [0.20]	-0.80 [0.14]	-0.50±0.16	-0.65±0.22	-0.644±0.094*	-0.733±0.091*	-0.334±0.097	-0.33±0.13
|E2A| (°)	42 [15]*	35.3 [5.3]*	27.2 [9.9]	23.1 [8.0]	59.4±5.4*	61.9±4.8*	15.7±5.6*	13.2±6.3*
E1 ($x10^{-3}$ mm^2s^{-1})	2.24 [0.18]*	2.13 [0.11]*	2.89±0.48*	2.49±0.33*	1.54 [0.16]*	1.417 [0.098]*	1.99±12*	1.92±0.14*
E2 ($x10^{-3}$ mm^2s^{-1})	1.58±0.11*	1.477±0.085*	2.03±0.34*	1.77±0.29*	1.041±0.081*	0.960±0.061*	1.040±0.075*	0.98±0.10*
E3 ($x10^{-3}$ mm^2s^{-1})	1.189±0.090*	1.10±0.11*	1.44±0.22*	1.27±0.22*	0.553 [0.064]	0.565 [0.043]	0.423 [0.056]*	0.441 [0.052]*
